# FTL004, an anti-CD38 mAb with negligible RBC binding and enhanced pro-apoptotic activity, is a novel candidate for treatments of multiple myeloma and non-Hodgkin lymphoma

**DOI:** 10.1186/s13045-022-01395-0

**Published:** 2022-12-29

**Authors:** Guangbing Zhang, Cuiyu Guo, Yan Wang, Xianda Zhang, Shuang Liu, Wen Qu, Chunxia Chen, Lingli Yan, Zhouning Yang, Zhixiong Zhang, Xiaohua Jiang, Xiaofeng Chen, Hong Liu, Qinhuai Lai, Xian Wei, Ying Lu, Shengyan Zhao, Han Deng, Yuxi Wang, Lin Yu, Hongbin Yu, Yu Wu, Zhaoming Su, Pengyu Chen, Ziqing Ren, Meng Yu, Feng Qu, Yong Luo, Lantu Gou, Qing Li, Ying Huang, Fanxin Ma, Jinliang Yang

**Affiliations:** 1 State Key Laboratory of Biotherapy and Cancer Center/Collaborative Innovation Center for Biotherapy, West China Hospital, Sichuan University, 3-17 People Road, Chengdu, Sichuan 610041 People’s Republic of China; 2 Sound Biopharmaceuticals Co., Ltd., Tianfu International Bio-Town, Huigu Dong 2nd Road 8, Chengdu, Sichuan 610200 People’s Republic of China; 3 Department of Clinical Laboratory, Mianyang Central Hospital, Mianyang, People’s Republic of China; 4 Department of Transfusion, West China Hospital, Sichuan University, Chengdu, People’s Republic of China; 5 Department of Clinical Research Management, West China Hospital, Sichuan University, Chengdu, People’s Republic of China; 6 Department of Hematology, West China Hospital, Sichuan University, Chengdu, People’s Republic of China; 7 Department of Head and Neck Oncology, West China Hospital, Sichuan University, Chengdu, People’s Republic of China; 8 West China School of Public Health, Sichuan University, Chengdu, People’s Republic of China; 9 Research Unit of Gene and Immunotherapy, Chinese Academy of Medical Sciences, Chengdu, People’s Republic of China

**Keywords:** Multiple myeloma, Monoclonal antibodies, CD38, Red blood cells, Direct apoptosis

## Abstract

Anti-CD38 monoclonal antibodies (mAbs), daratumumab, and isatuximab have represented a breakthrough in the treatment of multiple myeloma (MM). Recently, CD38-based mAbs were expected to achieve increasing potential beyond MM, which encouraged us to develop new anti-CD38 mAbs to meet clinical needs. In this study, we developed a novel humanized anti-CD38 antibody, FTL004, which exhibited enhanced pro-apoptotic ability and negligible binding to red blood cells (RBCs). FTL004 presented a better ability to induce direct apoptosis independent of Fc-mediated cross-linking against lymphoma and MM cell lines as well as primary myeloma cells derived from MM patients. For instance, FTL004 induced RPMI 8226 cells with 55% early apoptosis cells compared with 20% in the isatuximab-treated group. Of interest, FTL004 showed ignorable binding to CD38 on human RBCs in contrast to tumor cells, even at concentrations up to 30 μg/mL. Furthermore, with an engineered Fc domain, FTL004 displayed stronger antibody-dependent cellular cytotoxicity (ADCC) against CD38+ malignant cells. In vivo MM and non-Hodgkin lymphoma tumor xenograft models showed that FTL004 possessed an effective anti-tumor effect. Cryo-electron microscopy structure resolved two epitope centers of FTL004 on CD38: one of which was unique while the other partly overlapped with that of isatuximab. Taken together, FTL004 distinguishes it from other CD38 targeting mAbs and represents a potential candidate for the treatment of MM and non-Hodgkin lymphoma.

## To the editor

CD38 is a type II transmembrane glycoprotein with multiple functions and expressed lower on normal blood cells and higher on hematologic tumor cells [1, 2]. Anti-CD38 mAbs were effective in some hematological malignancies, especially multiple myeloma (MM) [3]. Although both daratumumab and isatuximab have significantly improved the outcome of patients with MM, incomplete responses and on-target/off-tumor effects emerge during the treatment [4, 5]. Herein, we described FTL004, a novel humanized IgG1κ anti-CD38 mAb possessing novel properties.

**Fig. 1:**
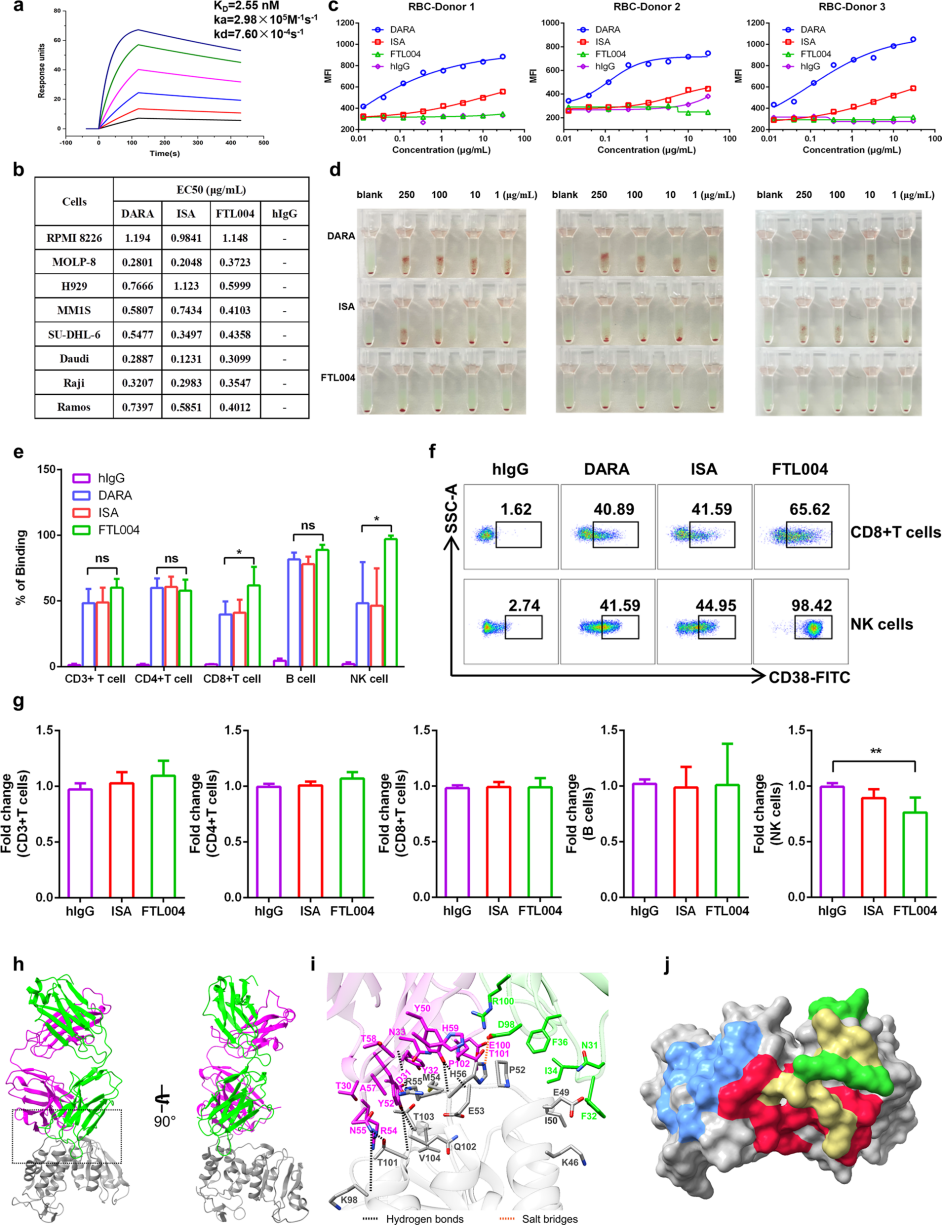
Distinct binding profile of FTL004 to CD38+ tumor cells, RBCs, and immune cells. **a** Affinity analysis of FTL004 to human CD38 as measured by Biacore 8 K (KD = 2.55 × 10^–9^ mol/L). **b** EC50 values for anti-CD38 mAbs binding to diverse cells. DARA, daratumumab; ISA, isatuximab. **c** Binding of FTL004, daratumumab or isatuximab to freshly isolated RBCs from healthy donors. **d** Indirect antiglobulin tests for CD38 mAbs to the same three healthy donors RBCs above. **e** The percentage of binding of CD38 mAbs to normal PBMCs (*n* = 4), including to CD45+ CD3+ T cells, CD3+ CD4+ T cells, CD3+ CD8+ T cells, CD45+ CD19+ B cells and CD3-CD56+ NK cells. Data show means ± SD and unpaired Student’s *t* tests were used to determine the statistical significance between daratumumab versus FTL004. *P < 0.05, **P < 0.01 and ***P < 0.001. **f** Flow cytometry dot plots showing the binding of anti-CD38 mAbs to NK cells and CD8+ T cells. **g** PBMCs from normal donors (*n* = 6) were pretreated with 1 μg/mL FTL004 or isatuximab for 3 days. Flow cytometry was used to determine the percentage of CD3+ T cells, CD4+ T cells, CD8+ T cells, B cells, and NK cells in lymphocytes. Data were then normalized to isotype controls and fold changes (means ± SD) are shown. Isotype cells were used as controls in settings. **h** The overall structure of the CD38/FTL004 Fab complex. CD38 and the heavy and light chains of FTL004 are colored gray, purple, and green, respectively. **i** Stereoscopic view of the direct hydrogen bonds, salt bridge, in the interface between CD38 and FTL004. **j** Comparison of the binding of FTL004, daratumumab, and isatuximab. Surface representation of the epitopes of FTL004 (green), daratumumab (blue), isatuximab (red) and superposition part of FTL004 and isatuximab (yellow) on CD38 (gray)

FTL004 exhibited similar affinities to CD38 (KD of 2.55, 3.84, and 1.2 nM, respectively) in surface plasmon resonance (Fig. 1a) and to CD38-positive cell lines in flow cytometry (Fig. 1b), compared with daratumumab or isatuximab. However, these antibodies bound differently to blood cells. Daratumumab or isatuximab bound to RBCs to different extents from healthy donors, while the binding of FTL004 to RBCs was virtually undetectable (Fig. 1c). This property should result in a more favorable pharmacokinetic of FTL004 by circumventing binding to CD38 on circulating RBCs [6] and a better safety profile by minimizing side effects associated with RBC binding. Moreover, FTL004 did not agglutinate RBCs in indirect antiglobulin tests (Fig. 1d), which could avoid the interferences with blood transfusion that are observed in other anti-CD38 mAbs [7, 8].

For other immune cells, FTL004 bound to most subpopulation cells of peripheral blood mononuclear cells (PBMCs) in a comparable intensity with daratumumab or isatuximab (Fig. 1e). Unexpectedly, FTL004 exhibited a slightly higher binding to CD8+ T cells and a much higher binding to NK cells (Fig. 1f). These events reflected that the epitope of FTL004 binding on CD38 may be distinct. Although anti-CD38 mAbs require a high threshold for CD38 expression to induce cell death, we must be wary of its possible off-target effects. PBMCs coculture assays showed that there was no depletion in T and B cells, indicating the limited off-tumor toxicity of FTL004 (Fig. 1g). But as expected, NK cells, with higher expression of CD38, were reduced to an acceptable level [9].

Then, the CD38/FTL004 complex structure was determined using Cryo-electron microscopy at 3.86 Å resolution (Fig. 1h, i). Of note, there seem to be two epitope centers of FTL004, one of them includes from Pro52 to His56 and the other one is 91TQTV94. Superimposition of the CD38/daratumumab (PDB code 7DHA) complex and CD38/isatuximab (PDB code 4CMH) complex onto the CD38/FTL004 complex revealed that the epitopes of FTL004 share no common residue of CD38 with daratumumab, whereas one of the epitopes (91TQTV94) of FTL004 partly overlaps with that of isatuximab (Fig. 1j).

**Fig. 2:**
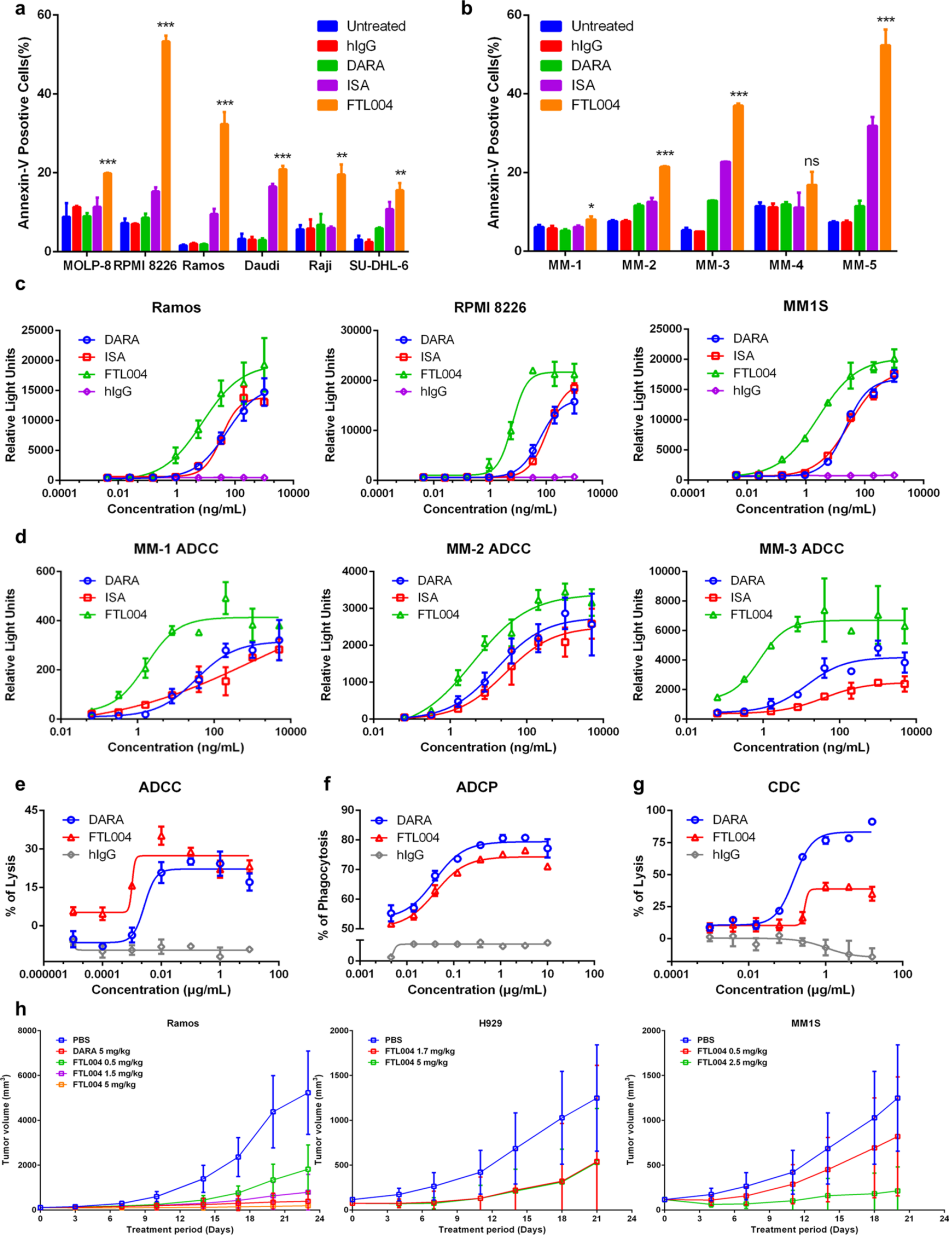
Anti-tumor efficacy of FTL004. **a** Induction of apoptosis by FTL004, daratumumab, or isatuximab in various cell lines, cells were treated with anti-CD38 mAbs 1.5 μg/mL for 24 h. Data represent means of triplicates and SD. Statistical significance between FTL004 and hIgG groups was determined by unpaired Student’s *t* tests. *P < 0.05; **P < 0.01, ***P < 0.001. **b** Apoptosis induction by FTL004 in primary MM cells. **c, d** ADCC reporter bioassays to various CD38+ tumor cell lines **(c)** and MM primary cells **(d)** of FTL004, daratumumab, or isatuximab. Data represent means of triplicates and SD. **e–g** Representative dose–response curve examples for ADCC activity **(e)**, ADCP activity **(f)**, and CDC activity **(g)** of FTL004 or daratumumab to CHO-CD38+ cells. Data show mean ± SD of three experiments. **h** Anti-tumor efficacy of FTL004 in lymphoma cell Ramos, MM cell H929, MM1S xenograft models. NOD-SCID mice bearing xenografts were treated with administration of PBS, daratumumab, or FTL004. Tumor burdens were monitored. Each point on the graph represents the average tumor volume

Next, to evaluate the tumor-killing capacity of FTL004, pro-apoptotic activity was first analyzed. We found that FTL004 had a superior ability to induce direct apoptosis independent of cross-linking reagents against CD38-positive cell lines (Fig. 2a) and primary MM cells (Fig. 2b) than isatuximab. Particularly, the highest apoptosis rate of FTL004 on primary MM cells was detected to be up to 60%. On the other hand, the Fc domain of FTL004 was engineered (S240D/I333E mutation) to elevate the affinity to human Fcγ receptors, leading to the enhancement of antibody-dependent cellular cytotoxicity (ADCC) [10]. In ADCC bioassays with Jurkat-Lucia-CD16a-NFAT cells [11], FTL004 showed a distinctly higher intensity of ADCC, with EC50 values of 2–8 ng/mL, compared with daratumumab and isatuximab (Fig. 2c). Consistent results were obtained in primary MM cells (Fig. 2d).

To investigate further, we examined the ADCC of FTL004 with healthy PBMCs. PBMCs exhibited enhanced ADCC activity against CHO-CD38+ cells in the presence of FTL004, with threefold higher than daratumumab (Fig. 2e). Meanwhile, FTL004 induced stronger ADCP with 74% phagocytosed CHO-CD38+ cells in an EC50 value of 40 ng/mL (Fig. 2f). However, FTL004 induced a lower CDC than daratumumab of CHO-CD38+ cells (Fig. 2g). In vivo studies were further carried out, and FTL004 was examined in Ramos, H929, and MM1S-bearing NOD-SCID mice (Fig. 2h). FTL004 treatment resulted in significant inhibition of tumor growth compared to PBS treatment.

In summary, FTL004 is a novel CD38 mAb that owns unique properties. Although the mechanism by which FTL004 binds differently to tumor cells versus RBCs remains unclear, it is expected that FTL004 owns limited on-target/off-tumor effects. The results above suggest that FTL004 is a therapeutic Ab with high potential for the treatment of MM and non-Hodgkin lymphoma.

## Data Availability

The datasets used and/or analyzed during the current study are available from the corresponding author upon reasonable request.

## Abbreviations

mAb: Monoclonal antibody
MM: Multiple myeloma
RBCs: Red blood cells
PBMCs: Peripheral blood mononuclear cells
ADCC: Antibody-dependent cellular cytotoxicity
